# Effects of psychological interventions and patients' affect on short‐term quality of life in patients undergoing colorectal surgery

**DOI:** 10.1002/cam4.739

**Published:** 2016-05-03

**Authors:** Gerold Koplin, Verena Müller, Grit Heise, Johann Pratschke, Wolfgang Schwenk, Oliver Haase

**Affiliations:** ^1^ Department of General, Visceral, Vascular and Thoracic SurgeryCharité ‐ University Medicine BerlinCampus MitteBerlinGermany; ^2^ Department of General and Visceral SurgeryAsklepios Klinik AltonaHamburgGermany

**Keywords:** Guided imagery, PANAS, progressive relaxation, quality of life, surgery

## Abstract

Psychological interventions can improve Quality of Life (QoL). Object of interest was if different psychological interventions influence short‐term QoL after colonic resection for carcinoma. Furthermore, we wanted to see if there is a correlation between patients` preoperative affect and postoperative QoL. Sixty patients that underwent colorectal surgery were divided into three groups. Group one (*n* = 20) received Guided Imagery and group 2 (*n* = 22) Progressive Muscle Relaxation. The third group (Control, *n* = 18) had no intervention. Quality of Life (QoL) was measured using the EORTC QLQ‐C30 and the Gastrointestinal Quality of life Index (GIQLI). Patients' affect was measured by the PANAS questionnaire. The higher the preoperative Negative Affect was, the lower were the scores for QoL on the 30th postoperative day. Patients' QoL was highest preoperatively and lowest on the third postoperative day. On the 30th postoperative day scores for QoL were almost as high as preoperative without difference between the three groups. Neither Guided Imagery nor Progressive Relaxation was influencing short‐term QoL measured by the EORTC QLQ‐C30 and the GIQLI questionnaire after colorectal surgery for cancer. Screening patients' with the PANAS questionnaire might help to identify individuals that are more likely to have a worse QoL postoperatively.

## Introduction

The impact of a therapy on quality of life (QoL) is important and may influence patient's choice of different therapeutic interventions. In non‐metastasized colonic cancer, surgical resection of the primary tumor should be performed whenever possible. Nonetheless surgery might affect QoL negatively. Sharma et al. examined QoL after colorectal surgery and speculated that preemptive interventions could improve postoperative QoL 1. It has already been demonstrated that psychological interventions have in fact a positive impact 2, 3. Tusek et al. were able to reduce postoperative consumption of analgesia and postoperative pain and increase patient comfort by using Guided Imagery 4. Moreover, QoL seems also to be affected by the patient's mood. Watson developed a score to measure patient's affect and distinguished between positive (PA) and negative (NA) affect 5. The idea of affect is that it is a stable and pervasive personality trait. NA is a predisposition to experience intense negative emotions. NA is related to stress and health complaints and high levels of NA are more likely to result in higher levels of distress and dissatisfaction 6, 7. Positive Affect reflect one`s level of pleasurable engagement with the environment. In patients with coronary heart disease health‐related QoL is associated with a person's level of PA 8.

The aim of this study was to examine the effect of different psychological interventions and patient's affect on short‐term changes of QoL.

## Methods

Quality of life was measured preoperative as well as on the third, seventh and 30th postoperative day using two questionnaires: First, the EORTC‐QLQ‐C30 (Version 1.0: Brussels, Belgium), a validated questionnaire created by the European Organisation for Research and Treatment of Cancer, containing 30 questions to assess QoL and second, the Gastrointestinal Quality of Life Index (GIQLI) by Eypasch 9, 10, 11, 12.

The questions or items of the QLQ‐C30 result in five functional scales (physical, role, cognitive, emotional, social), three symptom scales (fatigue, pain, nausea/vomiting) and several questions specifying symptoms such as dyspnea, loss of appetite, insomnia, constipation and diarrhea. The response categories are coded with four‐point Likert‐type scale: “Not at all”, “A little”, “Quite a bit” and “Very Much”.

The GIQLI questionnaire was developed by the surgeon Eypasch and contains 36 questions. There are five possible answers for each question, the most desirable option results in four points while the least desirable option results in zero points. Thus, the possible score of QoL ranges between 0 and 144 points with 144 points reflecting the best possible QoL.

The PANAS by Watson & Clark measures the PA and NA with two 10‐item mood scales. PA is composed by following descriptors: enthusiastic, interested, determined, excited, inspired, alert, active, strong, proud and attentive; NA by scared, afraid, upset, distressed, jittery, nervous, ashamed, guilty, irritable and hostile 5. A 5‐point Likert scale (“Not at all” = 1, “Very much” = 5) indicated the extent of the emotion. Thus, the range was 10–50 for each affect.

All patients above 18 years of age with a primary adenocarcinoma of the colon or rectum were screened for potential inclusion in the study. For inclusion, the planned procedure had to be open surgery to improve comparability. Excluded were all patients with an ASA‐Score > 3 (ASA, American Society of Anesthesiologists) or patients in an advanced stage of disease that would require extended surgery such as multi‐visceral resections. Furthermore, patients were excluded with a history of an abuse of analgesics or alcohol and chronic psychiatric or neurological disorders. Informed written consent was obligatory. The study was following the guidelines set by the Declaration of Helsinki and was approved by the Ethics Committee of the Charité University Medicine Berlin. The German Research Foundation (Deutsche Forschungsgesellschaft, DFG) funded the project.

Stratified Randomization was done 2 days prior operation. Stratification was due age (<60 years, 60–75 years or >75 years), gender and planned creation of an ostomy. Patients were either randomized in one of the two groups of intervention (Guided Imagery or Relaxation) or into the Control group.

Patients of the intervention groups obtained a portable audio player. The audible text for the Guided Imagery‐Group lasted 12 minutes and was based on Tusek's tutorials of Guided Imagery. It was supposed to help the patient reflecting and coping with feelings of anxiety and fear 4, 13.

An excerpt of the Guided Imagery text: *… You go on a journey… imagine a place where you feel safe and well… a place that calms you down and comforts you…*.

The text for the patients of the Relaxation‐Group lasted twelve minutes as well and contained text passages such *as …breath in slowly and deep, tense your muscles a little and then relax again…* The relaxation therapy was based on the concepts of Progressive Relaxation by Jacobson et al 14. The background music was the same for both groups.

Patients were supposed to hear the text three times daily, starting 2 days prior the operation. On the day of surgery, patients would hear no text but the same background music throughout the whole operation. The Control group did not have music or text at all.

After surgery, patients in the interventions group would continue to perform the exercises daily from the first postoperative day on for at least 30 days. The QLQ‐C30, the GIQLI and the PANAS Questionnaires were answered preoperatively and on the third, seventh and 30th postoperative day. Discharge from hospital was not before the seventh postoperative day. Patients took the audiotapes home to continue the exercises. Patients of the intervention groups would evaluate the experience of the exercises using an extra questionnaire. This questionnaire consisted of four questions (“Did you have the impression that you benefit from the exercises?”, “Was listening to the audio‐tapes pleasant?”, “Would you recommend other patients listening to the music before, during and after surgery?” and “Would you recommend other patients receiving surgery to use Imagery or Progressive Relaxation?”). Patients could answer “Yes”, “Rather yes”, “A bit”, “Rather No” and “No”.

Since data was collected as part of a larger study, the sample size was calculated assuming that a quarter less of the total intravenous morphine consumption in one of the interventions group would be a clinically significant difference 15. For this, at least 60 patients were needed. For statistical analysis *Statistical Analysis System* (SAS) 8.0^®^ for Windows 98^®^ (SAS Institute, Inc., Cary, NC) and SPSS (IBM SPSS Statistics for Windows, Version 21.0. Armonk, NY: IBM Corp) were used.

## Results

Within 23 months, 244 patients with colorectal carcinoma were treated in our department. 74 patients were eligible for the study. 13 patients were excluded because a laparoscopic procedure or a multi‐visceral resection was planned. One patient needed extensive surgery due to a secondary colorectal carcinoma that was diagnosed during operation. One patient withdrew his consent the day before surgery. One patient in the relaxation group could not listen to the audiotape on the second to third postoperative day due to a approximately 24 h lasting episode of a postoperative delirium.

Here 20 patients were in the Guided Imagery group, 22 in the Relaxation and 18 patients in the Control group. Of the 60 patients 23 (38%) were women and 37 (62%) men. 45 patients (75%) were operated due to a rectal carcinoma and 15 (25%) due to a colonic carcinoma. Half of the patients received a temporary loop ileostomy while in 11 patients there was the need to form a permanent colostomy. There were no significant differences between the groups concerning age, gender, body mass index, stage of disease, stoma formation during surgery or duration of the operation. There were no differences in the frequency of colonic or rectal cancer either. The patients' characteristics are shown in Table 1. A *P*‐value <0.05 indicated significance.

**Table 1:** Patients characteristics

	Imagery *n* = 20 Mean (SD)	Relaxation *n* = 22 Mean (SD)	Control *n* = 18 Mean (SD)	Total *n* = 60 Mean (SD)
Age (Years)	64.7 (8.6)	64.8 (9.9)	65.8 (11.5)	65.0 (11.0)
BMI (kg/m^2^)	27.9 (4.9)	26.2 (4.1)	28.1 (5.2)	27.4 (4.7)
Gender	*n (%)*	*n (%)*	*n (%)*	*n (%)*
Male/female	11/9 (55/45)	14/8 (64/36)	12/6 (67/33)	37/23 (62/38)
Stage (UICC)	*n*	*n*	*n*	*n*
0	3	1	1	5
I	7	9	5	21
II	4	6	6	16
III	6	6	6	18
Rectal resection	16	17	12	45
Colonic resection	4	5	6	15
Ostomy	16	13	12	41

UICC, Union internationale contre le cancer; BMI, Body mass index; SD, Standard deviation.

Preoperative scores for QoL measured by mGHS and GIQLI are in most groups higher than after surgery. In the Relaxation group, the postoperative GIQLI score on day 30 was higher than preoperative, but this was not significant (*P* = 0.25). Postoperative scores for QoL are lowest on the third and highest on the 30th postoperative day.

Analyzing the differences in the mGHS and GIQLI scores in all patients together over time using the paired *t*‐test, the measured decrease from preoperative to third postoperative day is in both scores significant (mean decrease: mGHS = 27.67, *P* < 0.0001; GIQLI = 8.40, *P* = 0.001). In the mGHS score, the difference between preoperative and the 30th postoperative day is significant as well, but not in the GIQLI score (mean difference: mGHS = −8.85, *P* = 0.014; GIQLI = −2.19, *P* = 0.287).

**Figure 1:**
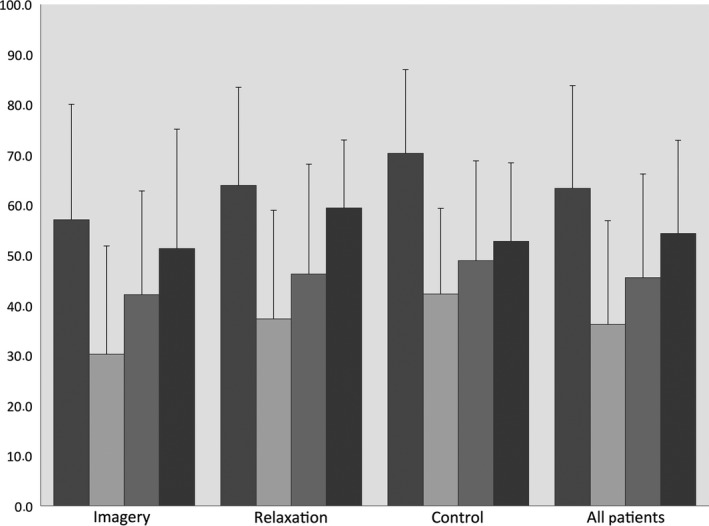
Perioperative changes in mGHS (Mean values). Left to right bar: Preoperative, 3rd, 7th, 30th postoperative day.

**Figure 2:**
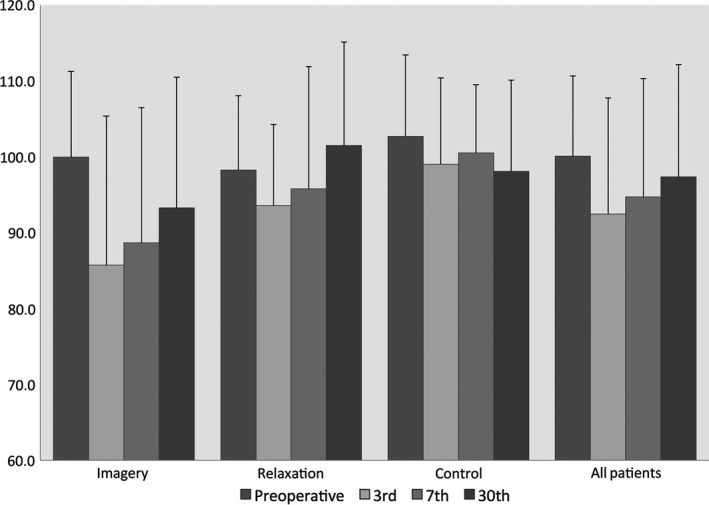
Perioperative changes in the Gastrointestinal Quality of life Indexscores (Mean values). Left to right bar: Preoperative, 3rd, 7th, 30th postoperative day.

There was a significant difference in the GIQLI score between the Imagery group (85.7) and the Control group (99.0) on the third postoperative day (*P* < 0.04). Other differences in that score were not detected (Figs. 1, 2).

The Physical and Role Function score in the Control group were higher on the seventh postoperative day than in the other groups on that day (*P* < 0.01). Cognitive Function score was higher in the Control on the third postoperative day (*P* = 0.027). Other differences between the groups on the different postoperative days in the Functional Scores could not be detected (*P* < 0.05, Table 2).

**Table 2:** EORTC QLQ‐C30 function and symptom scores in the different groups preoperative and on the 3rd, 7th, 30th postoperative day

		Imagery Mean (SD)	Relaxation Mean(SD)	*P* Imagery/Relaxation	Control Mean (SD)	*P* Control/Imagination	*P* Control/Relaxation
Function Scores							
Physical	Preop.	86.0 (23.5)	89.5 (20.6)	0.61	91.1 (24.9)	0.52	0.82
	3rd	37.0 (33.9)	39.0 (33.7)	0.85	55.6 (34.7)	0.11	0.14
	7th	51.0 (30.1)	41.9 (26.8)	0.31	67.8 (29.2)	0.09	**0**.**006**
	30th	64.2 (24.6)	73.0 (25.4)	0.28	72.2 (23.9)	0.32	0.92
Role	Preop.	80.0 (34.0)	76.2 (37.5)	0.73	91.7 (25.7)	0.25	0.15
	3rd	25.0 (38.0)	40.5 (43.6)	0.23	50.0 (42.0)	0.62	0.50
	7th	30.0 (41.0)	28.6 (37.3)	0.91	69.4 (38.9)	0.004	**0**.**007**
	30th	60.5 (39.4)	57.5 (40.6)	0.82	66.7 (29.7)	0.60	0.43
Symptom Scores							
Fatigue	Preop.	27.2 (18.9)	29.1 (19.7)	0.75	13.2 (14.8)	**0**.**02**	**0**.**01**
	3rd	80.2 (22.4)	69.4 (29.0)	0.29	60.4 (26.3)	**0**.**03**	0.34
	7th	63.2 (24.3)	64.3 (29.6)	0.90	46.5 (27.3)	0.07	0.07
	30th	56.8 (26.1)	56.6 (24.9)	0.98	48.1 (22.1)	0.32	0.33
Nausea/Vomiting	Preop.	1.7 (5.1)	3.2 (8.5)	0.50	1.0 (4.2)	0.70	0.37
	3rd	28.9 (25.4)	15.8 (17.5)	0.07	16.7 (25.8)	0.17	0.91
	7th	15.8 (26.3)	26.2 (35.6)	0,30	9.4 (12.1)	0.38	0.08
	30th	13.9 (21.6)	8.3 (19.2)	0.44	8.9 (10.7)	0.42	0.92
Pain	Preop.	9.2 (18.3)	21.4 (21.2)	0.55	4.2 (7.5)	0.31	**0**.**004**
	3rd	63.2 (32.7)	59.2 (26.8)	0.78	54.2 (26.2)	0.38	0.58
	7th	53.5 (32.2)	54.0 (24.1)	0.96	46.9 (23.7)	0.50	0.38
	30th	39.5 (29.5)	31.3 (25.0)	0.39	33.3 (32.1)	0.57	0.84
Dyspnoea	Preop.	13.3 (22.7)	11.1 (21.9)	0.72	4.2 (11.4)	0.15	0.26
	3rd	29.8 (33.1)	31.7 (29.6)	0.86	29.2 (24.0)	0.95	0.79
	7th	21.1 (27.7)	19.0 (22.5)	0.80	20.8 (31.9)	0.98	0.85
	30th	42.1 (33.0)	16.7 (21.1)	**0**.**01**	15.6 (27.8)	**0**.**02**	0.90
Appetite loss	Preop.	6.7 (23.2)	9.5 (18.7)	0.67	4.2 (16.7)	0.72	0.37
	3rd	77.8 (34.3)	56.7 (39.1)	0.09	45.8 (38.2)	**0**.**02**	0.41
	7th	57.9 (34.9)	49.2 (41.7)	0.48	31.3 (33.3)	**0**.**03**	0.17
	30th	31.5 (35.2)	27.1 (30.4)	0.70	22.2 (27.2)	0.41	0.64

*P* < 0.05 indicates significance (bold values).

SD, Standard deviation.

Looking at the Symptom scores, Fatigue, Nausea/Vomiting, Pain, and Dyspnoea scores were highest on the third postoperative day in all groups. The Imagery group had higher Appetite loss scores on the third postoperative day (*P* < 0.05) and higher Dyspnea scores on the 30th postoperative day than the other groups (*P* < 0.05). There were no other differences (Table 2).

**Figure 3:**
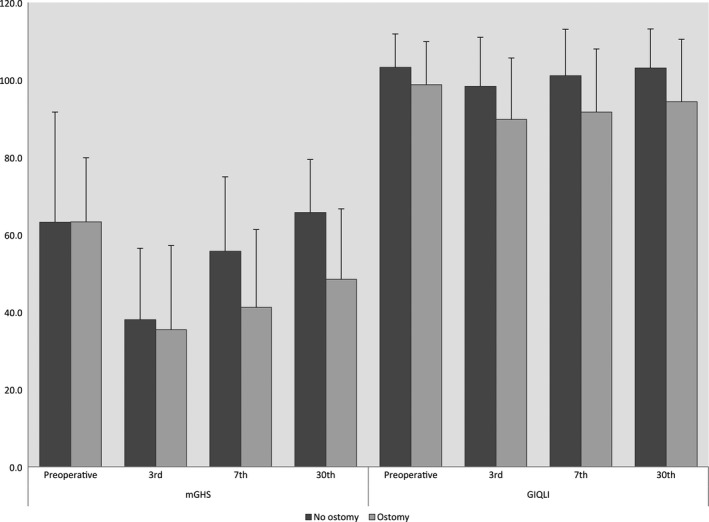
Perioperative changes in the mGHS (left) and Gastrointestinal Quality of life Indexscores (right; Mean values). Dark gray bar: Patients without an ostomy. Light gray bar: Patients with an ostomy postoperative.

The most notably impact on QoL had the creation of an ostomy. On day three after operation, the difference between nonostomy and ostomy patients were not significant, but on the 30th postoperative day the scores for QoL were distinctly lower in patients with an ostomy (Fig. 3). Patients in the Imagery group with an ostomy had a lower GIQLI score on the third postoperative day (79.92 vs. Relaxation = 94.21 and Control = 95.81; *P* = 0.017). Other differences or an ostomy‐independent effect of the psychological interventions could not be measured.

If groups were further split up and analyzed according to the procedure (right/left hemicolectomy, low anterior rectal resection), no difference could be detected either.

Analgesic dosage was recorded, but there was no difference between the groups concerning analgesic usage and there was no influence on Quality of Life (QOL).

Preoperative Negative Affect correlated negatively with the GIQLI‐score and mGHS‐score on the 30th postoperative day. The higher the preoperative NA was, the lower was the GIQLI‐ and mGHS‐score. Using Spearman‐Rho, the correlation coefficient for the correlation between preoperative NA and GIQLI on day 30 was *r *= −0.39 (*r* ^*2* ^= 0.15; *P* = 0.006) and between NA and mGHS was *r* = 0.34 (*r* ^*2* ^= 0.12; *P* = 0.018). No correlation was seen between QoL and PA.

Ninety percent of patients appreciated the interventions and would recommend them. Only 21% did not see an advantage in the interventions respectively the music with no difference between the groups either.

## Discussion

The level of negative affect, measured by the PANAS questionnaire, had an influence on QoL. The higher the preoperative NA was, the lower was QoL on the 30th postoperative day. This is in line with examinations on patients undergoing cardiac surgery. Patients experienced a lower QoL one and 6 month after surgery when preoperative NA levels were higher 16.

There were also changes in QoL after colorectal surgery, but neither Guided Imagery nor Progressive Relaxation had an advantage on short‐term changes of QoL measured by the EORTC QLQ‐C30 and the GIQLI questionnaires.

The lowest QoL was on the third postoperative day and recovered to almost the same level as preoperative on the 30th day after operation. The only difference between the groups measured by the GIQLI score was on the third postoperative day between the Imagery and the Control group. The score was significantly lower in the Guided Imagery group. It is not clear why the differences were in favor of the Control group. An explanation could be that the interventions lead to a more intense confrontation with the disease, which might affect QoL.

Interestingly, although we could not detect other statistical differences between the three groups, most of the patients in the interventions groups would not only recommend the psychological exercises but also see an advantage. The reason why patients in the Imagery group had a lower GIQLI score than in the Control group remains unclear.

The formation of an ostomy during surgery was very important for postoperative QoL. Though there was no difference between patients with or without ostomy on the third postoperative day, on the 30th postoperative day patients with an ostomy had a considerably lower QoL.

There have been attempts to influence QoL by psychological interventions 17, 18, 19. Our interventions received a positive feedback, but still we could not quantify a difference in QoL between the groups. Good had similar findings in 1995 20. The applied relaxation techniques after abdominal surgery had no significant effects on the level of pain that could be measured, but still most of the patients (89%) considered them helpful.

One questionnaire we used was the EORTC QLQ‐C30, a validated questionnaire which is used to assess health‐related QoL for over 20 years 9, 21. But still, there have been concerns that not all aspects of QoL are properly displayed by the EORTC questionnaire 22. Different questionnaires might have different emphases on different aspects of QoL. The EORTC questionnaire for example is focused on physical and less on psychological issues of QoL. To minimize the risk of missing an effect Cheung and colleagues suggested using more than one questionnaire 23. But even then, no difference could be seen using the GIQLI questionnaire additionally.

We did not examine long‐term effects. Interventions might take effect later than 30 days. Penedo examined 92 patients with prostate cancer who received either 10 weeks of group treatment (once a week for 2 h) or a single half‐day lasting training of strategies for coping with stress 24. QoL was assessed using the FACT‐G questionnaire. Only the men in the 10‐week treatment group experienced an improvement of QoL.

Most important for QoL though was not the intervention or the affect, but the existence of an ostomy postoperatively. On the third postoperative day there was no difference in QoL between the ostomy and nonostomy group. Apparently, patients have not realized until then the impact of the ostomy on their life, but 4 weeks after the operation they have. It was seen before and it is not unexpected that an ostomy influences QoL 25, 26.

We conclude from literature that there is evidence that QoL can be influenced by psychological interventions, which we could not prove in our setting. Screening for susceptible patients' might be helpful 27. But there are still miscellaneous variables, which have to be considered like using the appropriate questionnaires, applying the interventions long enough and measuring QoL at the right time after intervention. Data from literature is inconclusive and offer not enough guidance. Most important for QoL though is not the intervention but the existence of an ostomy. QoL is distinctively lower in patients with an ostomy.

Measuring preoperative NA might help to detect patients that are more likely to have a worse QoL postoperatively. Maybe through special care, outcome can be improved in those individuals.

## Conflict of Interest

None declared.
